# Comparison of Diagnostic Accuracy in Enteric Fever: The Widal Test, Advantage Typhi, and Typhiwell IgM ELISA Against the Gold Standard Blood Culture

**DOI:** 10.7759/cureus.101319

**Published:** 2026-01-11

**Authors:** Sadiya N Hajira, Ravi Giriyapur Siddappa, Rahil Pasha SA, Asmiya Parveen, Ruby Suresh Kumar Yadav, Raksha Yoganand

**Affiliations:** 1 Department of Microbiology, Sri Devaraj Urs Medical College, Kolar, IND; 2 Department of Microbiology, ESIC Medical College and PGIMSR, Bangalore, IND; 3 Department of Microbiology, Fortis Hospital, Bangalore, IND; 4 Department of Microbiology, Rao Tularam Memorial Hospital, New Delhi, IND; 5 Department of Microbiology, ESIC Medical College, Gulbarga, IND

**Keywords:** advantage typhi, blood culture, enteric fever, typhiwell-igm elisa, widal test

## Abstract

Introduction

Enteric fever remains a significant public health issue in developing countries, where timely and accurate diagnosis is crucial for appropriate therapy and infection control. This study aimed to evaluate the diagnostic performance of the Widal test and two rapid IgM-based assays (Advantage Typhi and Typhiwell IgM enzyme-linked immunosorbent assays (ELISA)) compared with blood culture, the gold standard.

Methods

A prospective study involving 410 clinical samples from patients with suspected enteric fever based on clinical findings was conducted between December 2015 and June 2017. Blood culture, the Widal test, and two rapid Salmonella IgM assays (Advantage Typhi and Typhiwell IgM ELISA) were performed. Sensitivity, specificity, positive predictive value (PPV), negative predictive value (NPV), and overall diagnostic accuracy were calculated.

Results

The Widal test demonstrated a sensitivity of 70.18%, a specificity of 74.22%, a PPV of 30.53%, an NPV of 93.91%, and an overall diagnostic accuracy of 73.66%. Advantage Typhi exhibited low sensitivity (12.28%) with moderate specificity (81.02%). The Typhiwell IgM ELISA showed the greatest sensitivity (88.46%) and the highest NPV (97.50%), but had reduced specificity (47.37%), leading to the highest overall diagnostic accuracy (95.89%).

Conclusions

The Typhiwell IgM ELISA provides improved diagnostic accuracy and may function as a rapid and effective diagnostic method in endemic regions. However, it should be used as a complementary test rather than a substitute for blood cultures. Additional studies, including molecular methods, are recommended to further enhance overall diagnostic accuracy.

## Introduction

Enteric fever, a potentially fatal multisystem infection, is caused by Salmonella Typhi (S. Typhi) (typhoid fever) and Salmonella Paratyphi (S. Paratyphi) (paratyphoid fever) [1]. Although typhoid fever has been largely controlled in developed countries through improvements in sanitation and water supply, it remains endemic in many parts of the world, particularly in the resource-limited regions of Asia, Africa, and South America [2]. Paratyphoid fever, caused by S. Paratyphi A, B, and C, is difficult to control and has distinct geographical distributions [3]. In India and other parts of Asia, S. Paratyphi A is predominant, whereas S. Paratyphi B and C are more common in Western Europe and parts of South America [4].

Enteric fever remains endemic in India, with studies reporting an incidence of 500 to 980 cases per 100,000 population [5]. The disease is most commonly observed in individuals aged 5 to 20 years [2]. Globally, it causes an estimated 22 million infections and 600,000 deaths each year, primarily in Asia [6]. Despite its high incidence, the diagnosis of enteric fever remains difficult because its clinical presentation overlaps with other febrile illnesses such as malaria, dengue, and viral enteritis [7]. Blood culture, which is the gold standard diagnostic method, has several limitations, including low sensitivity, a prolonged turnaround time, and the need for specialized laboratory facilities, making it less accessible in rural or resource-poor settings [2,7].

To address these challenges, alternative diagnostic approaches, including the Widal test, Typhidot, and newer rapid diagnostic tests (RDTs) based on immunochromatographic techniques (ICT) and enzyme-linked immunosorbent assays (ELISA), have been developed [8]. While the Widal test is widely used because of its cost-effectiveness and simplicity, it is limited by cross-reactivity, delayed antibody production, and poor specificity, leading to inaccurate results in certain settings [9]. More recently developed RDTs, such as Advantage Typhi and Typhiwell IgM ELISA, have shown promise in providing faster and potentially improved diagnostic results, although their diagnostic performance remains variable [10].

This study was designed to compare the diagnostic accuracy of the Widal test, Advantage Typhi (ICT), and Typhiwell IgM ELISA against blood culture in clinically suspected cases of enteric fever. By assessing sensitivity, specificity, and overall diagnostic performance, we sought to identify the most reliable and practical rapid diagnostic tool for enteric fever, particularly in resource-limited settings, where timely diagnosis is crucial for reducing morbidity and mortality.

## Materials and methods

Study design and setting

This prospective cross-sectional study was conducted at the ESIC Medical College and PGIMSR, Bengaluru, India, from December 2015 to June 2017 to evaluate the diagnostic accuracy of the Widal test, Advantage Typhi, an ICT, and Typhiwell IgM ELISA as compared to blood culture, which was considered the confirmatory test.

Study population

In total, 410 non-consecutive blood samples received from patients with clinically suspected enteric fever attending OPD or admitted to wards during the study period were included in the study.

Eligibility criteria

Inclusion Criteria

The inclusion criteria were as follows: blood and serum samples submitted to the microbiology laboratory for culture and serological testing, respectively, from patients with suspected enteric fever.

Exclusion Criteria

The exclusion criteria were as follows: samples with blood cultures showing mixed growth of more than one organism, indicating contamination; and samples from patients who tested positive for other infections.

Ethical consideration

Ethical clearance was obtained from the Institutional Ethical Committee (certificate number: 532/L/11/12/Ethics/ESICMC & PGIMSR/Estt.Vol. II)

Blood culture

Blood samples were inoculated into the BacT/ALERT PF bottle for samples from pediatric cases and the BacT/ALERT FA Plus bottle for samples from adult patients. These were incubated in the automated blood culture system BacT/ALERT 3D for a maximum period of seven days. Those that did not show any growth after seven days of incubation were taken as negative for growth. Bottles flagging positive were sub-cultured on blood and MacConkey agar plates. Identification of bacterial growth was performed using conventional biochemical tests: indole, mannitol, motility, citrate, urease, and triple sugar iron agar tests. Identification of Typhoidal Salmonella strains was confirmed by serotyping using specific antisera.

Widal test

The tube agglutination method was performed using the serum sample as per the manufacturer's instructions. Patient's serum was serially diluted from 1:20 to 1:640 in doubling dilutions using normal saline as the diluent. Four such sets of tubes were taken for which the O-, TH-, AH-, and BH-antigen suspensions were added, respectively. After overnight incubation at 37°C, Tubes showing about 50% agglutination with clearing were taken as positive. Titers of ≥1:80 for "O" and ≥1:160 for TH/AH/BH were considered significant. Samples showing significant titres in O and TH/ AH/ BH were considered positive.

Rapid diagnostic tests

Two rapid Salmonella-IgM tests, the Advantage Typhi IgM and IgG Card (J. Mitra & Co., New Delhi, India) and Typhiwell-IgM ELISA (AB Diachem Systems, New Delhi, India) were used.

Advantage Typhi IgM and IgG Card

It is a rapid, solid-phase ICT for the qualitative and simultaneous detection of S. Typhi IgM and IgG antibodies in human serum or plasma. The test was performed using serum samples according to the manufacturer’s instructions, with one drop of the patient’s serum and two drops of buffer added to the sample well of the test cassette. The result appeared as a colored line on the test bands within 20 minutes. Only the Salmonella IgM antibody results were evaluated in this study.

Typhiwell IgM ELISA 

It is an ELISA for the detection of IgM antibodies specific to S. Typhi in human serum/plasma. Out of 410 samples, 273 randomly selected samples were subjected to Typhiwell IgM ELISA. The test was performed in batches from serum samples stored at -20 °C, as per the manufacturer's instructions, using an automated ELISA washer. Interpretation was performed by reading OD values using an ELISA reader. Cut-off value was calculated for each run, and samples with values above the cut-off value were considered positive, and those less than the cut-off value were taken as negative.

Diagnostic performance evaluation

The test results were analysed using Microsoft Excel, and sensitivity, specificity, positive predictive value (PPV), negative predictive value (NPV), and diagnostic accuracy were calculated using blood culture as the gold standard.

Statistical analysis

Statistical significance was determined using chi-square tests, with a p-value <0.05 considered significant. Receiver operating characteristic (ROC) curve analysis was also performed to compare the diagnostic accuracy.

## Results

A total of 410 patients with clinically suspected enteric fever were included in this study. The demographic characteristics demonstrated a slight male predominance, with males comprising 56.34% (231 cases) compared with females, who accounted for 43.66% (179 cases). Most cases were observed in the pediatric age group between zero and 18 years, representing 90.73% (372 cases), followed by individuals aged 19 to 45 years, who constituted 7.81% (n = 32), while the smallest proportion belonged to the age group between 46 and 60 years, accounting for 1.46% (n = six). The blood culture positivity rate was 13.90%, with 57 of 410 patients testing positive, including Salmonella Typhi in 53 cases and Salmonella Paratyphi A in four cases.

Among 410 patients, 57 cases (13.90%) were positive for enteric fever by blood culture. This relatively low positivity rate underscores the limitations of blood culture sensitivity, particularly in endemic areas and among patients who may have received prior antibiotic therapy. The Widal test yielded positive results in 40 cases (70.18%) among blood culture-positive patients. In contrast, 17 cases (29.82%) that were confirmed by blood culture tested negative by the Widal test. This difference was statistically significant, with a p-value <0.05 (Table 1).

**Table 1:** Comparison of widal test wiith blood culture TP = 40, TN = 262, FP = 91, and FN = 17. Yates corrected chi-square test value = 42.47, p<0.0000001 TP: true positive; TN: true negative; FP: false positive; FN: false negative

Test result	Blood culture-positive (n = 57)	Blood culture-negative (n = 353)	Total
Widal positive	40 (70.18%)	91	131
Widal negative	17 (29.82%)	262	279
Total	57 (100%)	353	410

Using blood culture as the gold standard, the Widal test demonstrated a sensitivity of 70.18% (CI = 56.60% to 81.57%) and a specificity of 74.22% (CI = 69.32% to 78.71%). The positive predictive value was 30.53% (CI = 25.60% to 35.96%), while the negative predictive value was 93.91% (CI = 91.15% to 95.84%). Overall diagnostic accuracy was calculated to be 73.66%.

The Advantage Typhi test was positive for seven (12.28%) of blood culture-positive cases. However, it was negative in 50 (87.72%) cases that were culture positive for enteric fever. This was found to be statistically insignificant with a p-value >0.05 (Table 2). The Advantage Typhi test was negative for the four culture-confirmed paratyphoid fever cases.

**Table 2:** Comparison of Advantage Typhi with blood culture TP = 7, TN = 286, FP = 67, FN = 50. Yates corrected chi-square test value = 1.071, p = 0.1513 TP: true positive; TN: true negative; FP: false positive; FN: false negative

Test results	Blood culture-positive (n=57)	Blood culture-negative (n = 353)	Total
Advantage Typhi-positive	7 (12.28%)	67	74
Advantage Typhi-negative	50 (87.72%)	286	336
Total	57 (100%)	353	410

Using blood culture as the gold standard, the Advantage Typhi test showed a sensitivity of 12.28% (CI = 5.08% to 23.68%) and a specificity of 81.02% (CI = 76.53% to 84.98%). The PPV was 9.46% (CI = 4.81% to 17.77%), while the NPV was 85.12% (CI = 83.68% to 86.45%). The diagnostic accuracy of the Advantage Typhi test was determined to be 71.46%.

Typhiwell IgM ELISA yielded positive results in 23 cases (86.96%) that were confirmed by blood culture. However, the test was negative in three cases (13.04%) that were culture positive for enteric fever. This difference was statistically significant, with a p-value <0.05 (Table 3). Typhiwell IgM ELISA was also positive in two cases in which blood culture showed growth of Salmonella Paratyphi A.

**Table 3:** Comparison of Typhiwell IgM ELISA with blood culture (n = 273) TP = 23, TN = 117, FP = 130, FN = 3. Yates corrected chi-square test value = 10.85, p = 0.0004946 TP: true positive; TN: true negative; FP: false positive; FN: false negative

Test results	Blood culture-positive	Blood culture-negative	Total
Typhiwell IgM-positive	23 (86.96%)	130	153
Typhiwell IgM-negative	3 (13.04%)	117	120
Total	26 (100%)	247	273

Using blood culture as the gold standard, Typhiwell IgM ELISA demonstrated a sensitivity of 88.46% (CI = 69.85% to 97.55%) and a specificity of 47.37% (CI = 41.00% to 53.80%). The PPV was 15.03% (CI = 71.32% to 95.94%), while the NPV was 97.50% (CI = 93.03% to 99.13%). The diagnostic accuracy of this test was 95.89% (Table 4).

**Table 4:** Comparison of Widal, Advantage Typhi, and Typhiwell IgM ELISA with blood culture Chi-square test value = 29.3607, p = 0.00000123

Test results	Widal test	Advantage typhi test	Typhiwell IgM ELISA
Sensitivity	70.18%	12.28%	88.46%
Specificity	74.22%	81.02%	47.37%
Positive predictive value	30.53%	9.46%	15.03%
Negative predictive value	93.91%	85.12 %	97.50 %
Diagnostic accuracy	73.66%	71.46%	95.89%

The Widal test showed a moderate sensitivity (70.18%) and specificity (74.22%). The PPV and NPV were 30.53% and 93.9 1%, respectively, resulting in a diagnostic accuracy of 73.66%. The chi-squared test (p < 0.0001) indicated a statistically significant association with blood culture outcomes. (Tables 1, 4) The Advantage Typhi test demonstrated low sensitivity (12.28%) and moderate specificity (81.02%). PPV and NPV were 9.46% and 85.12 %, respectively, yielding a diagnostic accuracy of 71.46%. The chi-square test (p = 0.4914) showed no significant association with the blood culture results (Tables 2, 4).

The Typhiwell IgM ELISA demonstrated high sensitivity (88.46%) but lower specificity (47.37%). The PPV and NPV were 15.03% and 97.50 %, respectively, resulting in a diagnostic accuracy of 95.89%. The chi-squared test (p = 0.0008) indicated a significant association with blood culture results (Tables 3, 4). The highest sensitivity was shown by Typhiwell IgM ELISA at 88.46% followed by the Widal test and Advantage Typhi test with a sensitivity of 70.18% and 12.28% respectively. The highest specificity was shown by Advantage Typhi with a value of 81.02%, followed by the Widal test and Typhiwell IgM ELISA, showing specificities of 74.22% and 47.37% respectively.

**Figure 1:**
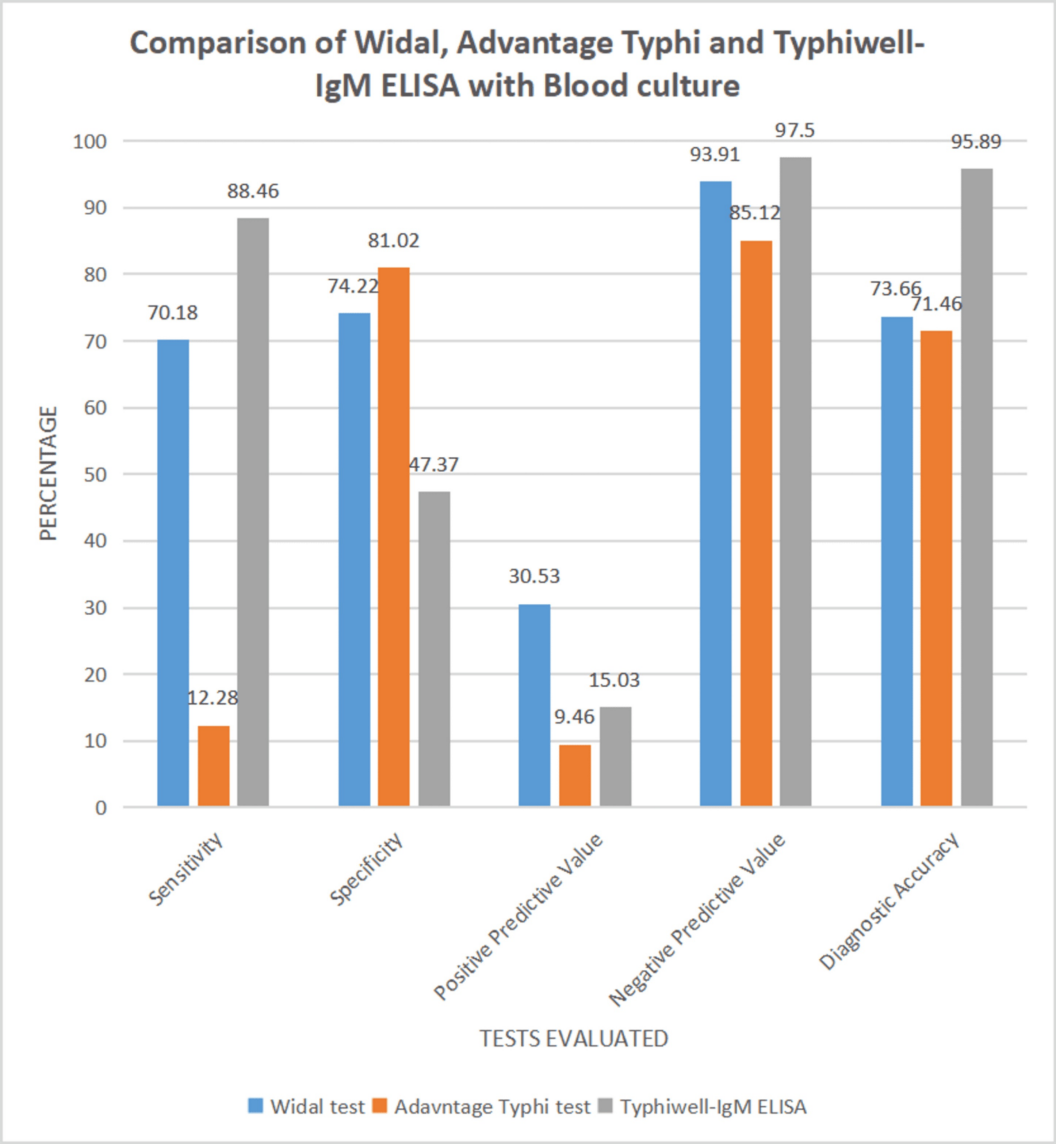
Comparison of Widal, Advantage Typhi, and Typhiwell IgM ELISA with blood culture

The Widal test had the highest PPV of 30.53%, followed by the Typhiwell IgM ELISA, having a PPV of 15.03% and the Advantage Typhi test with a PPV of 9.46%. Typhiwell IgM ELISA had the highest NPV of 97.50% (CI = 93.03% to 99.13%). This was followed by the Widal test with an NPV of 93.91% (CI = 91.15% to 95.84%) and the Advantage Typhi test with an NPV of 85.12% (CI = 83.68% to 86.45%) (Figure 1).

Overall, the Typhiwell IgM ELISA had the diagnostic accuracy of 95.89%, followed by the Widal test at 73.66% and the Advantage Typhi test at a value of 71.46%.

**Figure 2:**
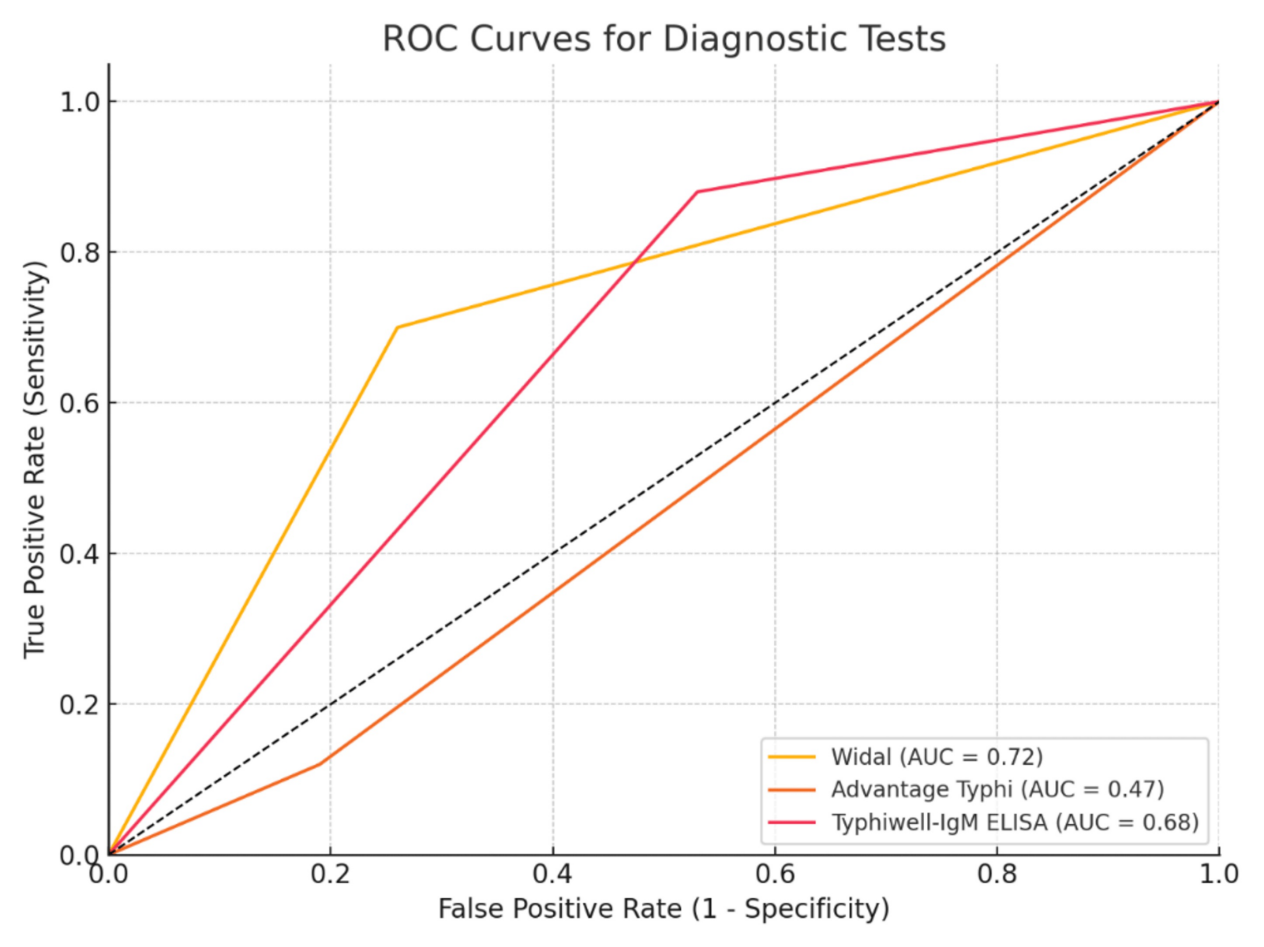
ROC curve comparison of diagnostic tests ROC: receiver operating characteristic; AUC: area under the curve

ROC curve analysis confirmed that the Typhiwel lgM ELISA exhibited the highest diagnostic accuracy, followed by the Widal test and the Advantage Typhi test, which showed significant limitations, particularly in sensitivity. The ROC analysis further demonstrated that the Typhiwell-lgM ELISA was the most reliable test for diagnosing enteric fever in this cohort (Figure 2)

## Discussion

This study evaluated the diagnostic accuracy of the Widal test, Advantage Typhi (ICT), and Typhiwell IgM ELISA in comparison with blood culture, the gold standard for diagnosing enteric fever. Enteric fever remains a significant health issue, particularly in endemic areas where early diagnosis is crucial for reducing morbidity and mortality. The study population predominantly consisted of individuals aged 0-45 years, which is consistent with previous studies that showed a higher prevalence of enteric fever in this age group. The majority of cases in our study were in the 0-18 years age range, mirroring findings by Saha et al. and Parande et al., who reported a higher incidence in the 0-20 years age group [11,12]. Olsen et al.’s study further supports the observation that enteric fever predominantly affects children and young adults [13].

A slight male predominance (56.34%) was noted in our study, which is consistent with the findings of Olsen et al. (56.96%) and Udaykumar et al. (56.30%) [13,14]. However, sex differences in enteric fever incidence are generally not significant, as also observed by Islam et al. (52.2%) [15]. Blood culture confirmed enteric fever in 13.9% of cases, a yield comparable to that reported by Garg et al. (13%) but lower than that reported by Udaykumar et al. (30.4%) [14,16]. Factors such as prior antibiotic use, small sample volumes (particularly in children), and late sampling likely contributed to the lower positivity rate. Most culture-positive cases (82.46%) were in the 0-18 years age group, consistent with the studies by Parande et al. and Saha et al., which reported a similar pediatric predominance [11,12].

The Widal test showed moderate sensitivity (70.18%) and specificity (74.22%), with a high NPV of 93.91% but a low PPV of 30.53%. These results are consistent with those of Andualem et al., Ngueguim et al., and Balakrishna et al. [17-19]. While the Widal test is commonly used in endemic areas, its diagnostic reliability is limited owing to cross-reactivity and prior exposure, warranting cautious interpretation. The Advantage Typhi IgM test demonstrated low sensitivity (12.28%) and moderate specificity (81.02%), which limits its diagnostic utility. This poor performance can be attributed to factors such as early testing, prior antibiotic use, and cross-reactivity in endemic areas. Although other IgM-based tests have shown better accuracy, this test is not suitable as a standard diagnostic tool. Since it only detects Salmonella Typhi IgM, it remained negative in culture-confirmed cases of paratyphoid fever, which further limits its utility in enteric fever diagnostics.

Typhiwell IgM ELISA exhibited the highest sensitivity (88.46%) and diagnostic accuracy (95.89%), making it a promising tool for rapid screening. The lower specificity (47.37%) was offset by a high NPV (97.50%). These findings are consistent with those reported by Bukhari et al. (100%), Nafi et al. (80%), and Khoharo et al. (88.5%) [20-22]. The Typhiwell IgM ELISA outperformed the Widal and Advantage Typhi tests in terms of both sensitivity and diagnostic accuracy. It was positive even in culture-confirmed cases of paratyphoid fever. Hence, it is a promising tool in the diagnosis of enteric fever. However, it cannot replace blood culture as the gold standard, and further studies are required to validate these findings.

The Widal test showed moderate concordance with the blood culture results, consistent with its long-standing use in the diagnosis of enteric fever. However, its performance can be influenced by factors such as prior Salmonella exposure, vaccination, and timing of testing. The Advantage Typhi test, with its poor concordance, has limited utility in diagnosing enteric fever. Conversely, the Typhiwell IgM ELISA showed good concordance with blood cultures, supporting its potential as a reliable diagnostic tool, although caution is needed because of its lower specificity. ROC curve analysis confirmed the diagnostic accuracy of the tests, with Typhiwell IgM ELISA showing the best performance. Although the Widal test demonstrated moderate accuracy, its lower sensitivity and susceptibility to false positives diminished its utility. The Advantage Typhi test, with its low sensitivity, was the least accurate, emphasizing its limitations in clinical practice.

Limitations of the study

Although this study provides valuable insights into the diagnostic performance of these tests, it has certain limitations. The blood culture positivity rate was relatively low, which may not fully reflect the true prevalence of enteric fever in the population. In addition, the study was conducted in a single-centre setting, and the results may not be generalizable to other regions or populations. Further multicentre studies with larger sample sizes are needed to validate these findings and improve the generalizability of the results.

## Conclusions

The Widal test and Typhiwell IgM ELISA showed reasonable diagnostic accuracy. However, the low sensitivity of the Advantage Typhi test limits its diagnostic effectiveness. Of the tests evaluated, the Typhiwell IgM ELISA demonstrated the highest diagnostic accuracy and could serve as an efficient rapid screening tool for enteric fever. However, it should not be considered as a substitute for blood culture, which remains the gold standard. Further research incorporating molecular methods like PCR for detecting Typhoidal Salmonella can provide confirmatory results against which diagnostic accuracy can be calculated. This approach can address the low sensitivity of blood cultures and generate more robust data on the comparative performance of the tests under study. Multicentric studies of this nature are needed to develop and strengthen recommendations for typhoid fever diagnostics in endemic regions.
